# Modelling and Analysis of the Positioning Accuracy in the Loading Systems of Mobile Cranes

**DOI:** 10.3390/ma15238426

**Published:** 2022-11-26

**Authors:** Wojciech Kacalak, Zbigniew Budniak, Maciej Majewski

**Affiliations:** 1 Faculty of Mechanical Engineering, Koszalin University of Technology, Racławicka 15-17, 75-620 Koszalin, Poland; 2 Faculty of Mechanical Engineering and Ship Technology, Gdańsk University of Technology, Narutowicza 11/12, 80-233 Gdańsk, Poland

**Keywords:** mobile cranes, loading systems, cargo handling systems, cargo transportation, positioning accuracy, precision kinematic systems, CAD/CAE, stability

## Abstract

In this work, the authors analyse the influence of the order and range of sequential movements of a crane’s working members on the accuracy of the final cargo positioning. The analysis was conducted on the basis of a specially developed method in which the authors proposed the introduction of a geometrical indicator of positioning the load in the intermediate positions (after completing each movement sequence) and in the target position, depending on the adopted control strategy and the accuracy of kinematic input of the working mechanisms (powered mechanisms). A mathematical model was presented to enable the accuracy of unidirectional positioning of the crane’s working members when conducting sequential movements controlled through the rotation of the crane column, inner and outer boom, and retractable stages of the six-section telescopic boom. Sample results of the numerical simulations showing the influence of the assumed kinematic inputs of the crane members on the accuracy of unidirectional angular and linear positioning and, consequently, on the accuracy of the final positioning of the transported cargo, were presented. Moreover, an indicator of the cargo positioning accuracy dependent on the location of an operator or a video camera and the trajectory of the cargo was developed, allowing the formulation of application conclusions.

## 1. Introduction

Previously, the authors of this study have worked on the problem of interactive systems containing a voice interface for the operator controlling crane equipment [1,2,3,4,5,6] using augmented reality [1,2], interactive systems [2,3], artificial intelligence [3,4], and antipatterns [5,6]. In such systems, voice signals [2,3,4] are sequentially transformed into formalized decisions resulting from the assumed movement strategy [6,7] and the evaluation of positioning [8,9], conditioning the correction or completion of a sequence of orders within the cargo handling task under realization.

To ensure the safety and efficiency of the cargo handling tasks conducted with the aid of cranes, including mobile cranes, it is possible to use various monitoring systems. These can include, but are not limited to, LIDAR scanners [10,11], interactive virtual reality systems for evaluating operations [12], numerical models for task visualization and trajectory tracking [13], as well as risk assessment methods for crane activities [14].

Recent studies have also reported progress on smart controllers [15], adaptive control [16], control strategies [17], stability simulations [18,19], heavy lifting modelling [20], and load carrying dynamics analysis [21].

Other challenging research tasks include stability analysis with swinging payloads [22], outrigger loads determination [23], movement trajectory optimization [7], heavy lift path planning [24,25], lift planning automation [26,27], and computational approaches for load positioning [28,29].

Kacalak, Budniak, and Majewski [30,31] proposed a stability assessment method of the mobile crane handling system with the use of a mathematical model and a model built in the integrated CAD/CAE environment. The article [32] described a computer-aided analysis using computational intelligence methods for the analysis and simulation research of a crane system during sequential movements. A parametric solid model was specified, designed with a CAD/CAE environment, which allowed the researchers to evaluate its stability for the selected configurations and conditions of operation.

Fang et al. [33], with the goal of improving the operators’ situation awareness, proposed a novel framework and practical system architecture for an operator-assistance system by leveraging real-time motion sensing and 3D modelling of dynamic workspaces. Yang et al. [34] proposed a synchronous control theory of the upper operation and the chassis attitude maintenance. The results of their research contributed to improving the safety of the truck crane, the operating efficiency, and reducing the operating burden of the driver. Hu et al. [35] reported mathematical models for selected practicality considerations in crane path planning. They proposed a safety-aware path planning method for single crane lifting scenarios. They validated their proposed algorithm in real lift tasks. The impact of lift task complexity on planning outcome was evaluated. They reported that an improvement on lift visibility and reduction of motion coordination were observed.

Cho and Han [36] proposed reinforcement learning for automated lift planning of cranes. They formulated mechanical and operational properties of lifting for a crane agent. Six modelling strategies were tested to train the RL agent in a virtual environment. They reported that the proposed approach was promising for planning feasible lifting paths and estimating reasonable lifting times, which helped generate and review lifting plans given the site conditions. Zhu et al. [37] presented an automatic crane-lift path planning system that was developed for modular integrated construction. The proposed platform was demonstrated to be effective and informative in simulating various crane lifts. Zhu and Pan [38] established a theoretical foundation for automated crane-lift path planning and contributed to the application of metaheuristics in the construction industry. Zhou et al. [39] proposed an improved RRT algorithm for lifting path planning of mobile cranes. The outcomes of the research contributed to the body of knowledge in spatial path planning of crane lifting operations, and also had the potential of significantly improving the efficiency and accuracy in crane lifting practices.

It is essential to achieve a high quality control of the crane’s operational movements to ensure the highest accuracy of positioning the cargo in the selected target point, minimizing the cargo fluctuation at the same time [40,41].

The issues connected with final positioning of the cargo handled by cranes were discussed, among others, in refs. [7,41,42,43], and concerned the cargo handling tasks realized with the aid of a rotary crane and positioning of the cargo after completing the rotary movement of the crane. In order to conduct a quantitative assessment of the positioning accuracy, two indicators were used. The first of them is based on the geometrical conditions, while the second one is based on the energy conditions [41]. The conducted numerical simulations confirmed their applicability for the analysis of the cargo positioning accuracy. To facilitate its quantitative assessment, the authors proposed a methodology using an integrated CAD/CAE system. The basic elements of the applied method include:

- Parametrical modelling of parts and components of the crane system in a CAD system for a defined configuration;
- Defining a geometrical indicator of the cargo positioning accuracy in the target position, depending on the assumed control strategy and accuracy of the working (powered) mechanisms’ kinematic input;
- Defining the accuracy of unidirectional angular and linear positioning of the crane’s working members using the example of a rotating column and retractable multi-stage telescopic boom (transcription of the equations constituting a mathematical model);
- Developing a numerical application and parametrical sketch in CAD-SolidWorks software in order to determine the accuracy of unidirectional positioning, depending on the position and perception of an operator conducting a visual assessment of the cargo location and the value of the parameters of the crane components’ operational movements;
- Developing a simulation model and numerical application to determine a geometrical indicator of the positioning accuracy, duration of the realization of the cargo handling task and distance covered by the cargo. The results of the numerical calculations—parameters of the operational movements—were introduced into the simulation model as input data;
- Analysis of the inaccuracy of a unidirectional angular positioning of the crane column and inaccuracy of the unidirectional positioning of a retractable multi-staged telescopic boom;
- Analysis of the accuracy of cargo positioning.

The conducted research consisted of simulating the operation of the crane for two instances of a conducted cargo handling task. In the first task, the influence of angular and linear dispersion of the crane’s unidirectional working members on the accuracy of cargo positioning was determined. In subsequent instances, the influence of the sequence of operational movements of the crane on the accuracy of the initial and final positioning of the cargo was analysed.

The article describes the application of two methods used for forecasting the accuracy of cargo positioning during the operation of hoisting equipment. One of the methods takes advantage of the possibilities offered by modern computing systems within an advanced engineering CAD/CAE software to build advanced simulation models, allowing an analysis and synthesis of the operation of hoisting equipment. In this case, the movements of the working members are defined in the CAD/CAE simulation model. The other one uses mathematical models containing explicit equations describing the dislocations of the working members of the cargo reloading system and allows determining the values of the indicators of positioning accuracy. Both methods applied for the analysis of the operation of the current and future hoisting equipment favour the complementarity of the assessment and better optimization of the trajectory of cargo displacement.

It is expected that the proposed methodology will contribute to widen the knowledge and practice, leading to a more efficient and safer operation of hoisting equipment supported by the operators of these devices. The proposed system leads to the improvement of the operators’ procedures and decisions. It fosters the improvement of the safety of operation of hoisting equipment, as well as a more efficient and precise realization of cargo handling tasks.

The developed method and simulation research can be useful in applying intelligent systems using voice command, augmented reality, and interactive systems. Moreover, the results of analyses of the proposed method provide the possibility of perfecting the strategy of cargo handling tasks and decision models for computer control systems for crane lorries.

Planning the trajectory of cargo displacement requires taking a sequential series of decisions, fulfilling at the same time the assumed criteria and limits. Previous research works concerning various automatic techniques of planning and optimization algorithms have been applied to facilitate more aware and optimized decisions regarding the realization of the cargo handling task.

The majority of research concentrates on individual planning decisions or a specific scenario of cargo displacement, which hinders the generalization and comparison of the results. Knowledge on the optimization of the cargo handling tasks is a collection of the assessment of completed processes and there is no connection between the operator’s observations and decisions and the accuracy of positioning. Taking the above into consideration, the authors developed synthetic indicators of cargo positioning accuracy, which include the repetitiveness and accuracy of the movement of crane members in the working space at each stage of the cargo handling task being realized, including the stability of the system. They also include the influence of the operator’s location in the working space on the positioning accuracy. The concept of replacing or supporting the operator with visual systems located in the vicinity of hooking the cargo has also been developed.

## 2. Accuracy of Positioning

**Figure 1:**
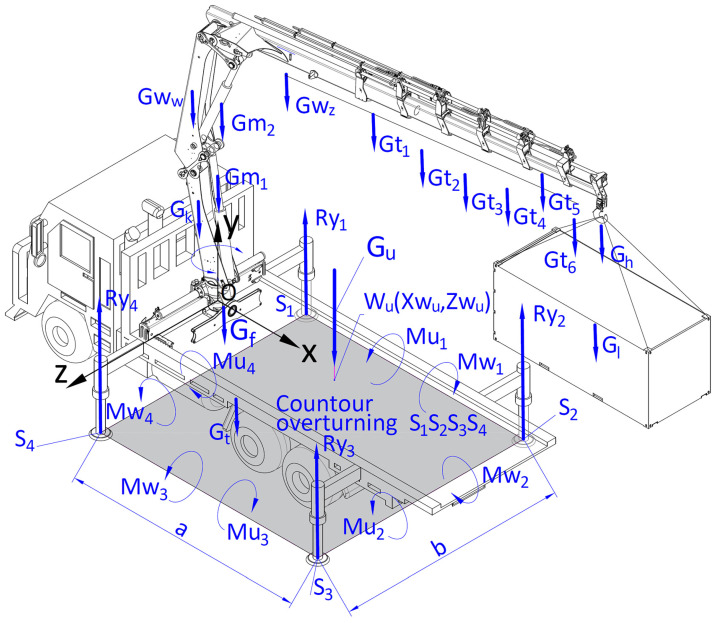
Calculation diagram for forces and torques acting on the crane outrigger system, where: $G_{u}$—total weight of the crane system; $G_{f}$—total weight of the truck with the outrigger system; $G_{b}$—weight of the crane base; $G_{k}$—weight of the rotating column; $Gw_{w}$—weight of the inner arm; $Gw_{z}$—weight of the outer arm; $Gm_{1}$, $Gm_{2}$—weights of the hydraulic actuators; $Gt_{1}$, $Gt_{2}$, …, $Gt_{6}$—weights of the arms of a six-member crane boom; $G_{h}$—hook weight; $G_{l}$—cargo weight; *a* and *b*—spacing of the crane outriggers.

The assessment of the positioning accuracy was preceded by the analysis of the cargo handling system’s stability (Figure 1) during the realization of a specific task. In order to do this, the following quantities were used:

- The location coordinates of the centre of gravity $S_{u}$ ($x_{Su}$, $y_{Su}$, $z_{Su}$) = f (t) of the cargo handling system. If the line of the action of the resultant of vertical pressure forces $G_{u}$ passes through $S_{u}$ point on the plane of the surface, defined by the coordinates $W_{u}\left(x_{Su},\;z_{Su}\right)$ and located inside the tipping contour $S_{1}S_{2}S_{3}S_{4}$, the crane is resting on all the outriggers in points $S_{1}$, $S_{2}$, $S_{3}$ and $S_{4}$;
- Vertical reaction forces $R_{1}$, $R_{2}$, $R_{3}$, and $R_{4}$ of the surface acting on the crane outriggers;
- The differences between the torques ΔM in relation to the tipping edges $k_{1}$, $k_{2}$, $k_{3}$ and $k_{4}$, described by Equations (1)–(4). In the calculations it was assumed, based on the ISO standard 4305:2014 [44], that the stability is ensured when the value of the differences between the torques ΔM is larger than 0, and it is calculated using the following dependency (1):
  (1) $$\begin{array}{c} \Delta M=min(\Delta M_{1},\Delta M_{2},\Delta M_{3},\Delta M_{4})>0 \end{array}$$
  where: $\Delta M_{1}$, $\Delta M_{2}$, $\Delta M_{3}$, $\Delta M_{4}$—the differences of the torques in relation to the tipping edges $k_{1}$, $k_{2}$, $k_{3}$ and $k_{4}$; where: $k_{1}=S_{1}S_{2}$, $k_{2}=S_{2}S_{3}$, $k_{3}=S_{3}S_{4}$, $k_{4}=S_{4}S_{1}$;
  (2) $$\begin{array}{c} \Delta M_{i}=Mu_{i}-Mw_{i} \end{array}$$
  (3) $$\begin{array}{c} Mu_{i}=\sum_{j=1}^{n}G_{j}\cdot d_{i_{j}} \end{array}$$
  (4) $$\begin{array}{c} Mw_{i}=\sum_{j=n+1}^{m}G_{j}\cdot d_{i_{j}} \end{array}$$
  where: $Mu_{i}$—setting torque; $Mw_{i}$—tipping torque; *j*—number of the members in the crane system; *n*—number of the members in the crane system whose vector of the gravity force $G_{j}$ during the cargo handling in the projection on a horizontal plane xz is located within the tipping contour limited with the edges $k_{1}$, $k_{2}$, $k_{3}$, and $k_{4}$; *m*—number of all the members of the crane, *i*=1 ÷ 4—number of the tipping edge, $d_{i_{j}}$—distance between the centre of gravity of the element *j* from the tipping edge *i* in the projection on the horizontal plane xz expressed as (5):
  (5) $$\begin{array}{c} d_{1_{j}}=z_{j}-z_{s_{1}},d_{2_{j}}=x_{s_{2}}-x_{j},d_{3_{j}}=z_{s_{4}}-z_{j},d_{4_{j}}=x_{j}-x_{s_{1}} \end{array}$$
  where: $x_{j}$, $z_{j}$—coordinates of the location of the *j* element in the projection on the horizontal plane xz;
  $x_{s_{1}}$, $x_{s_{2}}$—coordinates of the location of outriggers in the support points $S_{1}$ and $S_{2}$;
  $z_{s_{1}}$, $z_{s_{4}}$—coordinates of the location of outriggers in the support points $S_{1}$ and $S_{4}$.
- Safety indicator $W_{b}$, as a criterion of the crane system stability, defined as (6):
  (6) $$\begin{array}{c} W_{b}=min\in \left\{\frac{min(Ry_{n})}{G_{u}\cdot f_{1}\left(1-f_{2}\right)}\right\}-\frac{f_{2}}{1-f_{2}} \end{array}$$
  (7) $$\begin{array}{c} t=t_{e}-t_{b} \end{array}$$
  where *n* = 1 ÷ 4—number of an outrigger; $min(Ry_{n})$, kN—the lowest of the vertical reactions of the surface on an outrigger *n*; $G_{u}$, kN—total weight of the crane system; $f_{1}$—coefficient of the maximum loading of the crane outrigger; $Ry_{max}$ = $G_{u}\cdot f_{1}$, where $f_{1}\leq$ 0.25 for a crane with four outriggers; $f_{2}$—coefficient determining the minimum loading of the crane outrigger; $Ry_{min}$ = $G_{u}\cdot f_{2}$; *t*, *s*—duration of the work cycle of the cargo handling task; $t_{b}$ = 0, *s*—initiation of the work cycle of the crane; $t_{e}$, *s*—end of the crane’s work cycle.

In order to ensure the stability of the crane system, the value of the safety indicator $W_{b}$ should be larger than zero, when $min(Ry_{i})$>$f_{1}\cdot f_{2}$. In turn, the value of $f_{2}$ coefficient is determined, due to safety reasons, on the level depending on the working conditions of the crane. It was assumed that the value of this coefficient takes wind speed and speeds of acceleration, and tugging in kinematic pairs of the crane into consideration. Tugging may be caused by tearing off the cargo frozen to the surface, breaking the cargo loose, sudden braking, hitting an obstacle, etc.

**Figure 2:**
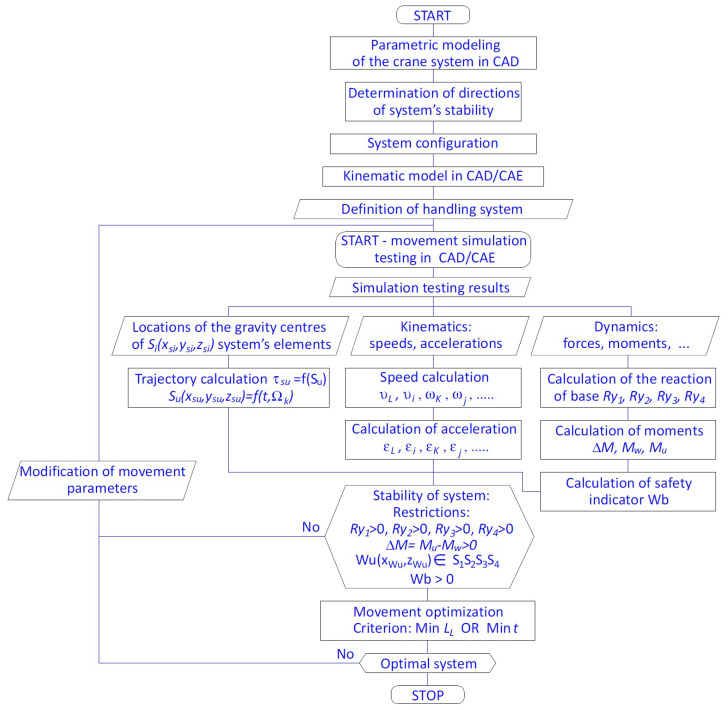
Block diagram of computer-aided assessment of the stability of the crane handling system [31].

The methodology [31], presented in Figure 2, was used in simulation testing to assess the stability of the mobile crane handling system. The simulation model built with the use of the integrated CAD/CAE system makes it possible to assess the stability of the crane system on the example of the HIAB XS 111 crane with the proposed interaction and control system [2,3].

The following are the basic elements of the implemented method:

- Parametric modelling of the elements and the entire crane system in the CAD system for the defined configuration;
- Determination of the system stability conditions (a notation of equations that constitute a mathematical model to calculate the following: the trajectory of the mass centres of the elements of the crane system, the reaction of the base on the crane outrigger system, the stabilizing torque Mu, and the overturning torque Mw as well as the safety indicator);
- Building of a kinematic model of the crane and carrying out simulation testing in the integrated CAD/CAE system;
- An analysis of the kinematic and dynamic quantities of the crane system during handling in connection with maintaining constant balance (stability);
- An optimization of the trajectories of the displacements of the working systems of the crane for specific assignments taking into consideration the movement safety indicator, also considering the limiting conditions. By knowing the value of the safety indicator during working movements, it is possible to conduct an assessment of the risk of the loss of the crane’s stability and to select the optimal displacement trajectory.

An integrated CAD–SolidWorks software as well as the module for computations and engineering analyses: CAE–SolidWorks Motion was used for the purpose of the modelling and numeric tests of the crane handling system.

**Figure 3:**
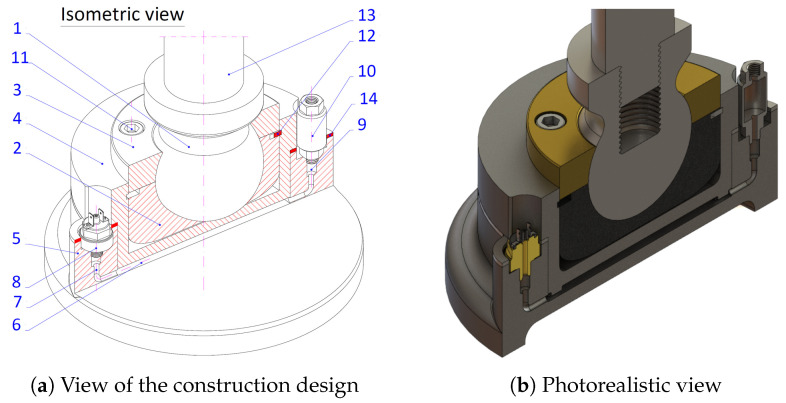
Crane outrigger design [45] with an incorporated pressure system and a reciprocal valve, where: 1—ball pin; 2—hemispherical bowl; 3—upper hemispherical bowl; 4—sleeve with a flange; 5—foot base, area filled with oil; 6—oil filled chamber; 7—duct; 8—pressure sensor; 9—duct; 10—reciprocal valve; 11—screw; 12—inner setting ring; 13—piston; 14—sealing washer.

In order to control the reaction at the outriggers, a special construction of the outrigger of a crane device was developed [45]. It is equipped with a hydraulic system measuring the pressure transferred by the outriggers, where the outrigger’s foot features an elastic sleeve with a membrane and incorporated pressure sensor, coupled with a measuring system that constitutes an integral part of the crane device system [46] (Figure 3).

The accuracy of positioning the cargo handled with a crane depends on the accuracy of the sequential displacement of its particular powered members. It was assumed that the accuracy of positioning is the difference between the distance between a set point, usually a point connected with the handled cargo, and the real point. In order to facilitate a quantitative assessment of the accuracy, the introduction of cargo positioning accuracy indicators was proposed.

It was assumed that the set accuracy of positioning the cargo in an intermediate position, after completing a single sequential movement and in the target position, can be achieved in two stages [47]:

1. Initial positioning—in the first stage of the cargo handling task, sequential movements with possibly high speed of powered members of the crane are conducted. Hence, it is to be expected that the accuracy of positioning the cargo in the intermediate points and in the target point shall be burdened with a relatively large positioning error;
2. Target (precise) repositioning—purposeful slowing down of the movements of the crane’s working members occurring near the target spot. The number of sequential movements and duration of the cargo repositioning will depend on the set accuracy of placing the cargo in the target position.

**Figure 4:**
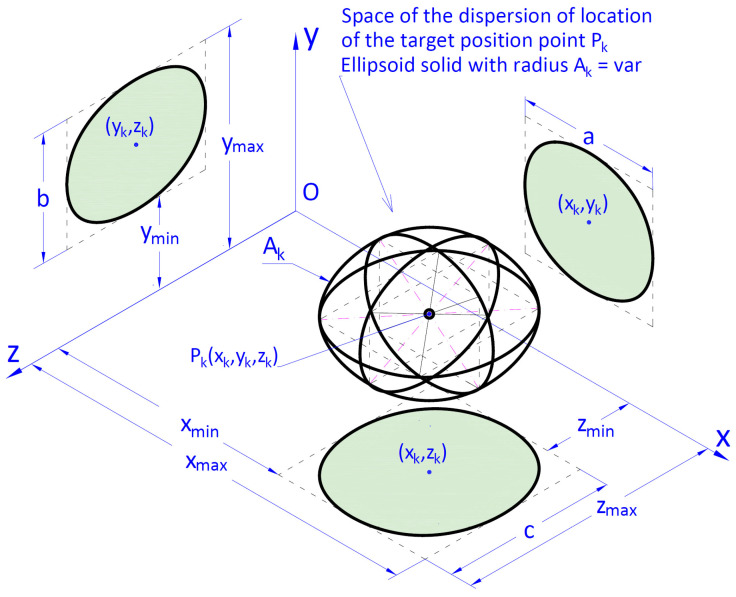
Geometrical interpretation of the positioning accuracy indicator $A_{k}$. Positioning accuracy of the handling crane when transporting the cargo from the start point $P_{p}$ to the given final point $P_{k}$.

Geometrical interpretation of the $A_{k}$ indicator is presented in Figure 4. It is a radius of an ellipsoid described on a cuboid whose sides are parallel to the axis of an absolute coordinates system describing the location of the target point after completing the cargo handling task.

It was assumed that the geometrical indicator of positioning accuracy $A_{k}$ (8) is the maximum value of the cargo location distance in relation to the nominal location $P_{k}=L_{k}\left(x_{Lk},\;y_{Lk},\;z_{Lk}\right)$:

(8) $$\begin{array}{cc} A_{k} & =r_{g}-r_{d}=\sqrt{{D_{x}}^{2}+{D_{y}}^{2}+{D_{z}}^{2}}=\\ \; & =\sqrt{{\left(\frac{x_{max}-x_{min}}{2}\right)}^{2}+{\left(\frac{y_{max}-y_{min}}{2}\right)}^{2}+{\left(\frac{z_{max}-z_{min}}{2}\right)}^{2}} \end{array}$$

where: $x_{max}$, $x_{min}$, $y_{max}$, $y_{min}$, $z_{max}$, $z_{min}$—are the respective maximum and minimum values of the coordinates of the final location of the cargo. However, the ranges of the values of coordinates of positions towards *x*, *y*, *z* are $D_{x}$, $D_{y}$, $D_{z}$; $r_{d}$, $r_{g}$—lower (9) and upper (10) absolute value of the vector of distance between the coordinates of the cargo position and beginning of the coordinates system:

(9) $$\begin{array}{cc} r_{d} & =|{\vec{r}}_{d}|=\sqrt{{\left(x_{L_{k}}-\frac{D_{x}}{2}\right)}^{2}+{\left(y_{L_{k}}-\frac{D_{y}}{2}\right)}^{2}+{\left(z_{L_{k}}-\frac{D_{z}}{2}\right)}^{2}} \end{array}$$

(10) $$\begin{array}{cc} r_{g} & =|{\vec{r}}_{g}|=\sqrt{{\left(x_{L_{k}}+\frac{D_{x}}{2}\right)}^{2}+{\left(y_{L_{k}}+\frac{D_{y}}{2}\right)}^{2}+{\left(z_{L_{k}}+\frac{D_{z}}{2}\right)}^{2}} \end{array}$$

where: $x_{L_{k}}$, $y_{L_{k}}$, $z_{L_{k}}$—coordinates of the vector ${\vec{r}}_{k}$ determining the nominal position of the cargo (required remote position) (11):

(11) $$\begin{array}{c} {\vec{r}}_{k}=L_{k}=\left[x_{L_{k}},y_{L_{k}},z_{L_{k}}\right],\\ x_{L_{k}}=\frac{x_{max}+x_{min}}{2},\\ y_{L_{k}}=\frac{y_{max}+y_{min}}{2},\;z_{L_{k}}=\frac{z_{max}+z_{min}}{2} \end{array}$$

The length of the vector $|{\vec{r}}_{k}|$ (12) is the distance of the nominal position of the cargo from the beginning of the system of coordinates Oxyz:

(12) $$\begin{array}{c} r_{k}=\left|{\vec{r}}_{k}\right|=\sqrt{{x_{L_{k}}}^{2}+{y_{L_{k}}}^{2}+{z_{L_{k}}}^{2}} \end{array}$$

**Figure 5:**
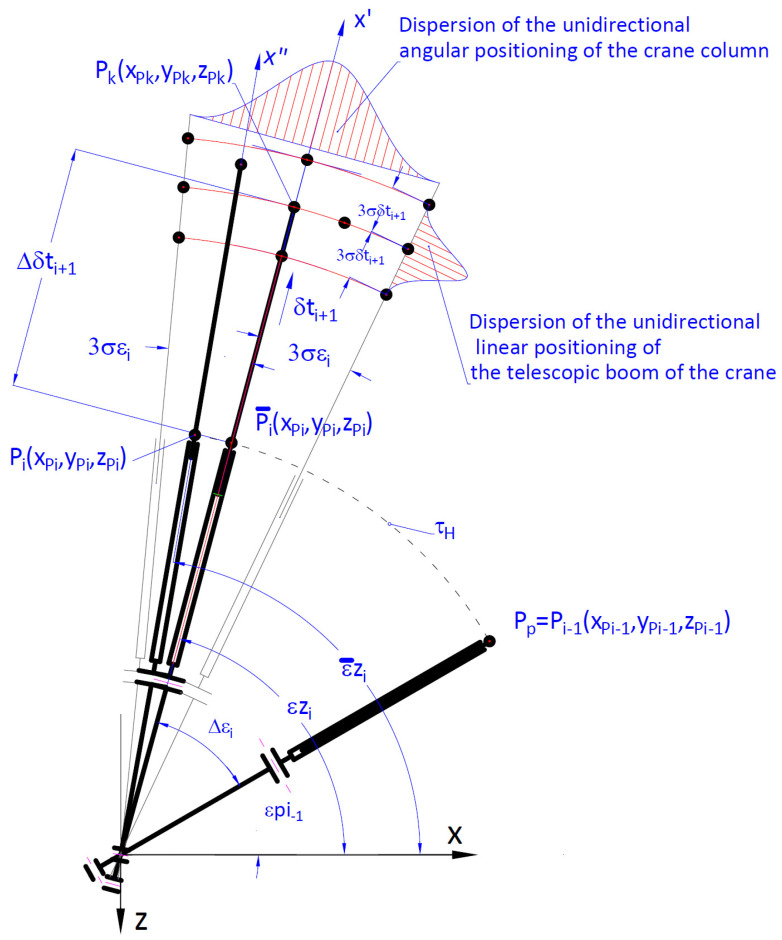
Diagram for calculating the accuracy of unidirectional positioning of the cargo handling crane: for transporting the cargo from the initial position $P_{p}$ to the set target position $P_{k}$, where: *x*—axis of the system of coordinates in relation to which the angular orientation (on the example of the rotation of the crane column) is measured; $x^{\prime }$—axis of the set angular orientation relative for the measurement of linear displacement (on the example of a retractable telescopic boom), *i*—subsequent sequential movement of the crane, $\varepsilon_{Z}i$—angle of the set position of $x^{\prime }$ axis, ${\bar{\varepsilon }}_{Z}i$—mean value of the angular orientations, $\delta t_{Zi+1}$—set position according to linear orientation, $\varepsilon_{Z}i$—mean value of the linear position, $\pm 3\sigma_{\varepsilon i}$—dispersion of the angles in relation to the values ${\bar{\varepsilon }}_{Z}i$, $\pm 3\sigma_{\delta ti+1}$—dispersion of unidirectional linear displacement in relation to the value $\bar{\delta }t_{Zi+1}$.

**Figure 6:**
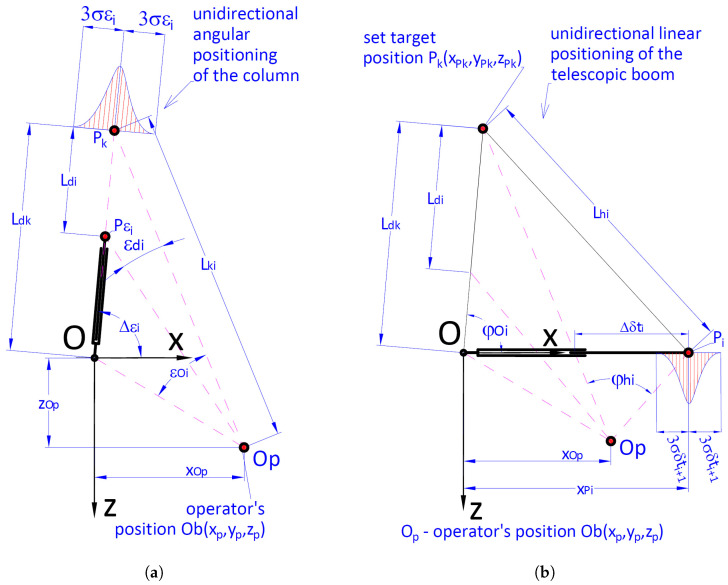
Parametrical sketch for determining the accuracy of unidirectional positioning of the cargo handling crane depending on an operator’s position Op($x_{Op}$,$z_{Op}$). (**a**) unidirectional angular positioning of the crane column. (**b**) unidirectional linear positioning of a telescopic boom.

Taking into consideration the working movements of the crane when reaching the set intermediate position, after completing a subsequent sequence of linear or rotary movement, the accuracy of unidirectional positioning for particular powered members can be determined as:

(A) Accuracy of the angular positioning of the rotary members of the cargo handling crane, defined on the basis of the calculation diagram presented in Figure 5.
  The defined value of the angular error (13) of unidirectional positioning of a rotary movement, on the example of the rotation of crane column in relation to the expected value ${\bar{\varepsilon }}_{z_{i}}$ is determined by the dependency:
  (13) $$\begin{array}{c} \Delta \varepsilon_{i}=6\cdot \sigma_{\Delta \varepsilon_{i}}=\Delta \varepsilon_{O_{i}}+\Delta \varepsilon_{t_{i}} \end{array}$$
  where: $\Delta \varepsilon_{O_{i}}$—accuracy of unidirectional estimation of the crane column’s angular position by an operator in intermediate position, expressed as (14)
  (14) $$\begin{array}{c} \Delta \varepsilon_{O_{i}}=C_{p_{\varepsilon }}\cdot C_{\varepsilon_{Oi}}\cdot \varepsilon_{di} \end{array}$$
  where: $\Delta \varepsilon_{t_{i}}$—accuracy of angular position resulting from the reaction time $t_{h}$ of an operator controlling the crane and angular velocity ${\dot{\varepsilon }}_{i}$ of the column, expressed as (15):
  (15) $$\begin{array}{c} \Delta \varepsilon_{t_{i}}=C_{m_{\varepsilon }}\cdot {\dot{\varepsilon }}_{i}\cdot t_{h}/2 \end{array}$$
  where: $C_{\varepsilon_{Oi}}$—coefficient of the accuracy of estimation of the telescopic boom’s angular position depending on the crane operator’s position, expressed as (16):
  (16) $$\begin{array}{c} C_{\varepsilon_{Oi}}=\frac{L_{di}\cdot L_{ki}}{{L_{dk}}^{2}}/\log_{10}\left(\varepsilon_{di}+10\right) \end{array}$$
  where: *i*—number of a subsequent sequential movement where *i* = 1÷n;
  *n*—number of sequential movements of the crane,
  $L_{di}$, $L_{ki}$, $L_{dk}$, $\varepsilon_{di}$—geometrical parameters depending on the position of the crane operator OP Op($x_{Op}$,$z_{Op}$). Values of these parameters were determined on the basis of a parametrical sketch developed in CAD (Figure 6a),
  $C_{P_{\varepsilon }}$—coefficient of the visual perception of an operator, defining his ability to correctly estimate unidirectional angular displacement of the crane’s working member,
  $C_{m_{\varepsilon }}$—mass coefficient of the accuracy of positioning of the rotation angle $\Delta \varepsilon_{i}$ (the speed of the angular displacement is reversely proportional to the mass of the handled cargo).
(B) Defined value of the error of unidirectional linear positioning of a slide movement of a multi-member telescopic boom of the crane, in relation to the expected value $\bar{\delta }t_{Zi}$ determined by the dependency (17):
  (17) $$\begin{array}{c} \Delta \delta t_{i}=6\cdot \sigma_{\Delta \delta t_{i}}=\Delta \delta t_{Oi}+\Delta \delta t_{ti} \end{array}$$
  where: $\Delta \delta t_{ei}$—unidirectional accuracy of the estimation of placing the cargo in the intermediate position by the crane operator, expressed as (18):
  (18) $$\begin{array}{c} \Delta \delta t_{O_{i}}=C_{P_{\delta t}}\cdot C_{\delta tO_{i}}\cdot L_{pi} \end{array}$$
  $\Delta \delta t_{t_{i}}$—error of the accuracy of linear positioning resulting from the reaction time $t_{h}$ of an operator controlling the movement of the crane, and linear velocity ${\dot{\delta t}}_{i}$ of the telescopic boom, determined as (19):
  (19) $$\begin{array}{c} \Delta \delta t_{t_{i}}=C_{m_{\delta t}}\cdot {\dot{\delta t}}_{i}\cdot t_{h}/2 \end{array}$$
  $C_{{\delta t}_{Oi}}$—coefficient of estimating the accuracy of unidirectional linear positioning of the slide $\Delta \delta t_{i}$, depending on the position of the crane operator, expressed as (20):
  (20) $$\begin{array}{c} C_{{\delta t}_{Oi}}=\frac{L_{hi}\cdot L_{ki}}{{L_{dk}}^{2}}\cdot \sqrt{\varphi hi} \end{array}$$
  $L_{hi}$, $L_{pi}$, $\varphi_{hi}$—geometrical parameters depending on the position of the crane operator $Op(x_{Op},z_{Op})$—Figure 6b;
  $C_{P_{\delta t}}$—coefficient of the visual perception of an operator, defining his ability to correctly estimate unidirectional linear displacement of working members of the crane;
  $C_{m_{\delta t}}$—mass coefficient of the accuracy of unidirectional linear positioning of the rotation angle (the speed of the linear displacement is reversely proportional to the mass of the handled cargo).

**Figure 7:**
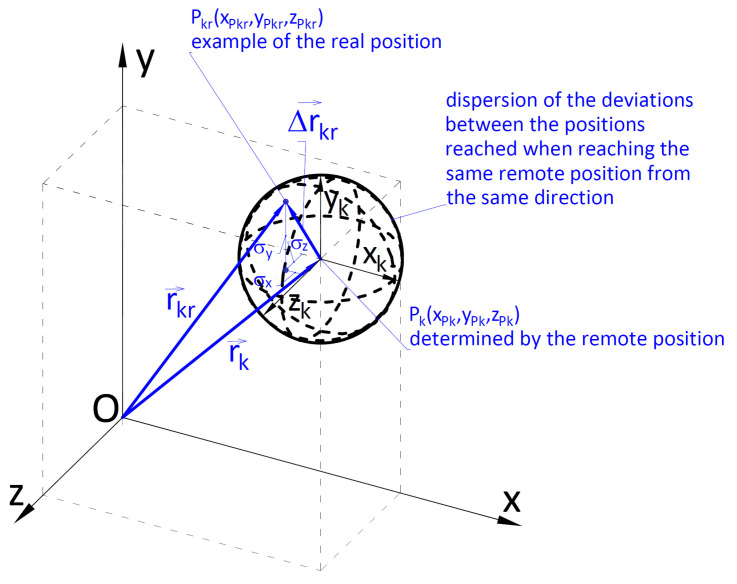
Accuracy of positioning the cargo handling crane when transporting the cargo from the initial position $P_{p}$ to the set target position $P_{k}$.

**Figure 8:**
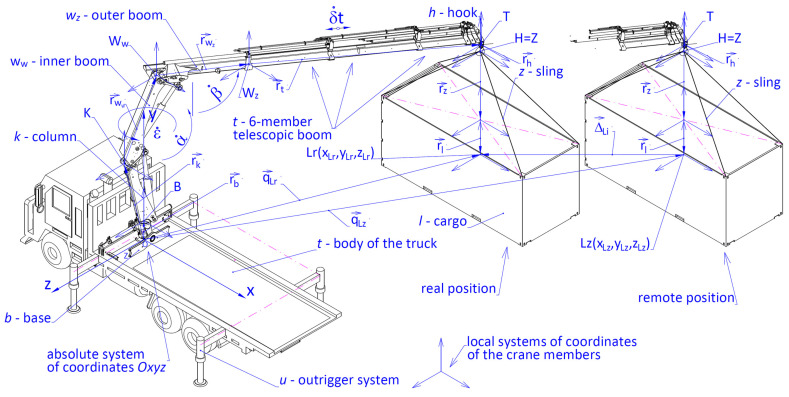
Spatial dimensional chain of the crane system where: ${\vec{\Delta }}_{kr}$—closing link determining the accuracy of positioning the cargo handling crane after completing *i*th sequential movement of the working member, ${\vec{r}}_{kr}$—vector determining the real position of the cargo, ${\vec{r}}_{k}$—vector determining the set (nominal) position of the cargo.

In order to ensure the required positioning of the cargo with a specific accuracy, it is, primarily, necessary to determine the mutual position of the members within the crane system. In a general case, the accuracy of the positioning of the handled cargo is determined by a vector $\Delta {\vec{r}}_{kr}$ (Figure 7 and Figure 8) which is the closing link of a spatial 3D chain.

In the absolute system of coordinates Oxyz, the closing link’s value is (21):

(21) $$\begin{array}{c} \Delta {\vec{r}}_{kr}={\vec{r}}_{kr}-{\vec{r}}_{k}=\left[\begin{array}{c} \Delta x_{kr}\\ \Delta y_{kr}\\ \Delta z_{kr} \end{array}\right]=\left[\begin{array}{c} x_{P_{kr}}-x_{P_{k}}\\ y_{P_{kr}}-y_{P_{k}}\\ z_{P_{kr}}-z_{P_{k}} \end{array}\right] \end{array}$$

where:

${\vec{r}}_{k}=P_{k}=\left[\begin{array}{c} x_{P_{k}}\\ y_{P_{k}}\\ z_{P_{k}} \end{array}\right]$—vector determining the location of the handled cargo’s required remote position,

${\vec{r}}_{kr}=P_{kr}$—vector determining the real position of the handled cargo.

The analytical description of the configurations of the crane’s kinematic system consists of transmutations of the vector-matrix equations [7,8]. The transmutations were conducted until clear dependencies determining variable angular and linear configuration values were obtained. Knowing such dependencies is extremely desirable. However, it should be emphasized that for the cargo handling system of a crane, obtaining clear dependencies is extremely tedious. In the light of the presented difficulties, a simulation model developed in an integrated CAD/CAE system was used to determine the vectors specifying the real position of the handled cargo and its positioning accuracy.

The simulation model allows determining the position of any element of the crane system during the realization of the cargo handling task. Hence, on the basis of the conducted research, it is possible to determine the value of ${\vec{r}}_{kr}=P_{kr}$ vector in the final position. Subsequently, it is possible to calculate the accuracy of cargo positioning using formula (21). By knowing the accuracies of cargo positioning $\left(\Delta x_{kr},\Delta y_{kr},\Delta z_{kr}\right)$ along each axis of the absolute coordinates system Oxyz, it is possible to calculate summary inaccuracy of the cargo positioning using formula (22):

(22) $$\begin{array}{cc} \Delta P_{k} & =\sqrt{{\left(\Delta x_{kr}\right)}^{2}+{\left(\Delta y_{kr}\right)}^{2}+{\left(\Delta z_{kr}\right)}^{2}}\\ \; & =\sqrt{{\left(x_{P_{kr}}-x_{P_{k}}\right)}^{2}+\left(y_{P_{kr}}-y_{P_{k}}\right){)}^{2}+{\left(z_{P_{kr}}-z_{P_{k}}\right)}^{2}} \end{array}$$

The calculated inaccuracy of cargo positioning should fulfil the condition (23):

(23) $$\begin{array}{c} \Delta P_{k}\leq A_{k} \end{array}$$

## 3. Simulation Model of the Cargo Handling System

In order to determine the accuracy of positioning in the simulation research, the authors used a simulation model of a cargo handling system fixed on a self-propelled crane HDS HIAB XS 111 type shown in Figure 8. To determine how the parameters of linear and angular displacements of the working members of the crane influence the positioning of its particular members in the initial, operating (working) and final positions, 3D nodes were introduced in the simulation model. In order to force a relative dislocation of the cargo for the needs of the movement simulation, two types of powered members were incorporated in the model. The first type included members conducting a rotational movement of the crane column with $\dot{\varepsilon }$ velocity. The second type included linear powered members forcing rotational movements of the inner and outer boom with $\dot{\alpha }$ and $\dot{\beta }$ velocities and progressive movement of a six-member telescopic boom with $\dot{\delta }t$ velocity.

## 4. Simulation Research

Conducted research consisted in the simulation of the crane operation for various configurations. In the first research, the influence of angular and linear dispersions of the crane’s unidirectional working members on the accuracy of cargo positioning was determined on the basis of the model constructed in the CAD/CAE system and calculating application.

**Figure 9:**
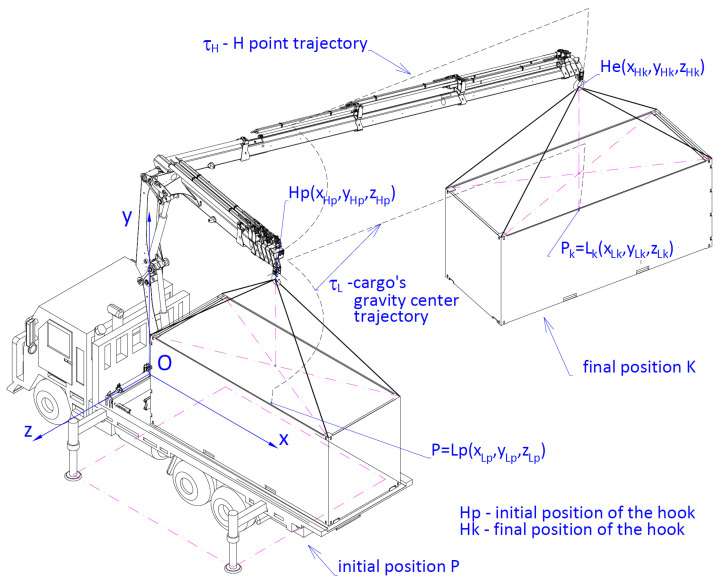
Working cycle of the crane’s cargo handling task.

In the presented example, for a cargo handling task shown in Figure 9, the configuration of the working movements of the crane during the realization of this task is listed in Table 1, where the notations of the position parameters have been assumed in compliance with Figure 8. Cargo weighing $Q_{l}$ = 800 kg located in the initial position *P* was supposed to be carried to the final position located in the $P_{k}$ point.

The results of the simulation research served as a basis for determining the influence of the compound inaccuracies of the angular positioning of the column’s rotation and linear retraction of the telescopic boom on the accuracy of positioning the cargo in its final position.

**Figure 10:**
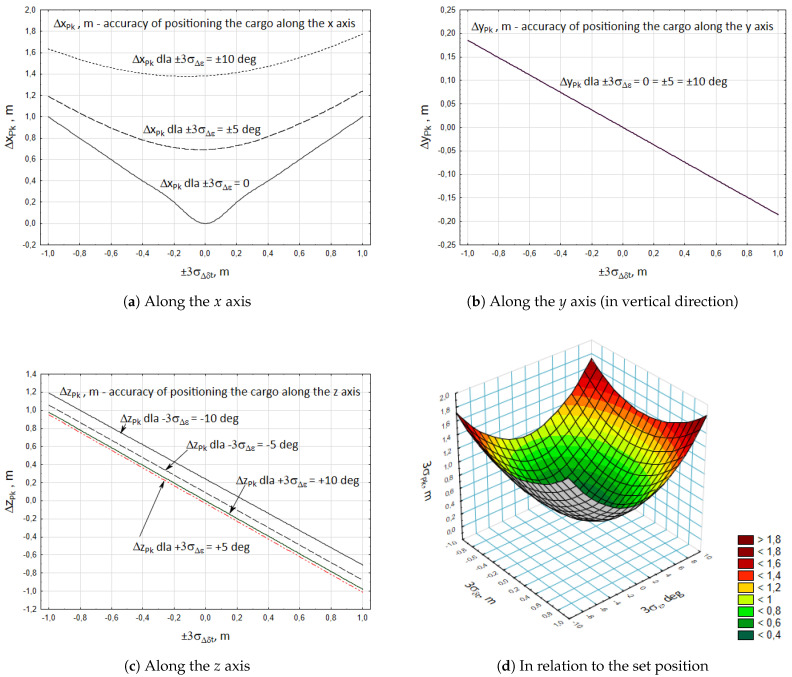
Accuracy of positioning the cargo in the target position.

Figure 10 presents the values specifying the dispersion of the inaccuracy of positioning the cargo along the particular axes of the absolute system of coordinates Oxyz of the crane. It also shows the summary dispersion of the cargo positioning in relation to the set target position.

Conducting the analysis, in the case of forcing a rotary movement of the crane column with the accuracy of ±3 $\sigma_{\varepsilon 2}=\pm$10 deg and retraction of the telescopic boom with the accuracy of ±3 $\sigma_{\delta t3}=\pm$1 m, a significant dispersion of positioning the cargo in horizontal plane was obtained. Maximum dispersion of positioning $\Delta x_{Pk}$ ranged from 0 to 1.62 m (Figure 10a). In the direction towards the z axis it was $\Delta z_{Pk}$ = −0.98 ÷ 1.19 m (Figure 10b). The dispersion of the crane cargo positioning was lowest in the vertical direction and it was $\Delta y_{Pk}$ = −0.19 ÷ 0.19 m (Figure 10c).

In turn, the summary dispersion of cargo positioning $\Delta P_{k}$ in relation to the set target position of the cargo ranged from 0 to 1.775 m.

The analysed example included the realization of the initial positioning in which the particular working movements are conducted with possibly high propulsion velocities. Hence, the result shows that the obtained positioning errors are quite significant.

The second example shows how the location of the crane operator who conducts a visual assessment of the cargo position influences the inaccuracy of unidirectional positioning of the crane’s working members. Consequently, it also influences the accuracy of initial positioning of the cargo.

The analysis was divided into two parts. In the first part, formulae (6 ÷ 13) and values of the parametrical sketch parameters (Figure 6) were used to calculate the inaccuracies of unidirectional angular positioning of the crane column and linear positioning of the multi-member telescopic boom. The analysis was conducted for two cases of the cargo handling task being realized. Sequences of the crane’s working movements and their nominal parameters have been listed in Table 2.

In both cases, it was assumed that the mean reaction time of the crane operator was $t_{h}$ = 1.25 s, the operator’s position was changing in the range of $x_{Op}$ = −3 ÷ 9 m, $z_{Op}$ = 2 ÷ 6 m, value of the mass indicator $Cm_{\varepsilon }$ = $Cm_{\varepsilon }$ = 1. The mean values of the angular and linear orientations reflect their set values. In the numerical calculations it was assumed that the working movements were possibly conducted with high velocities for the initial positioning. Rotary movement of the crane position was realized with the velocity of $\dot{\varepsilon }$ = 18 deg/s, linear propulsions forcing the rotary movement of the inner and outer boom with the velocities of $\dot{\alpha }$ = 5 deg/s and $\dot{\beta }$ = 2.5 deg/s, and retraction of the six-member telescopic boom with the velocity of $\dot{\delta }t$ = 0.3 m/s.

**Figure 11:**
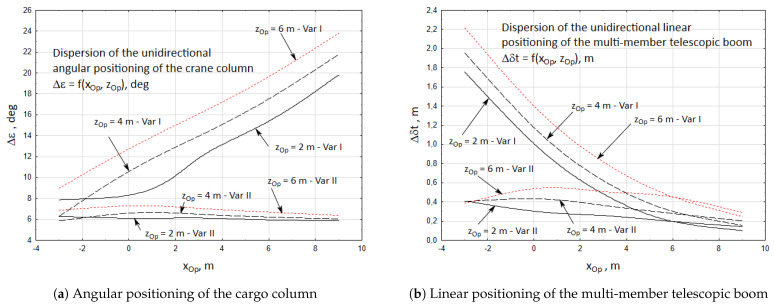
The accuracy of the initial unidirectional positioning of the cargo.

The results of the numerical calculations have been presented in Figure 11. The graphs show the relation between the operator’s position and the accuracy of unidirectional positioning of the crane’s working members.

The second part of the analysis consisted in conducting simulation research of the initial cargo positioning accuracy. Results of the numerical calculations presented in Figure 11 were adopted as the input data.

**Figure 12:**
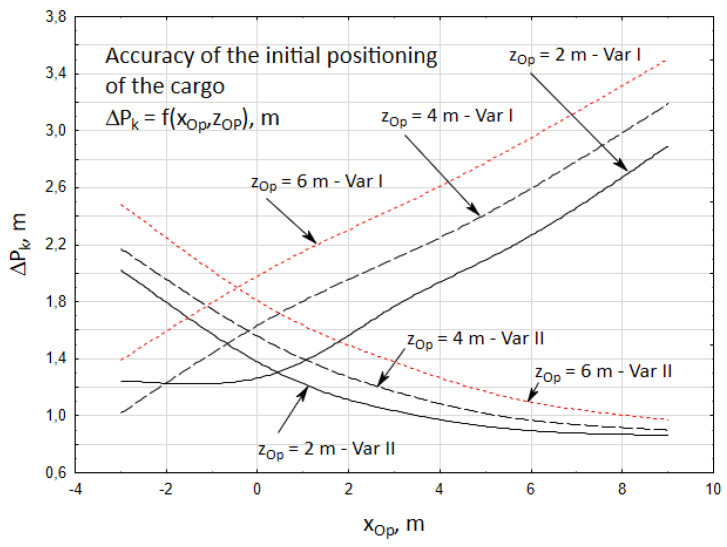
Accuracy of the initial positioning of the cargo $\Delta P_{k}$, for two cases of the cargo handling task.

The obtained results show (Figure 12) that the accuracy of the initial positioning of the cargo features a diversified nature, depending on the position of the crane’s operator.

It was observed that the accuracy of positioning the cargo, for both cases, is higher when the operator of the crane is closer to the vehicle towards the *z* axis. As the operator moves away from the vehicle along the *z* axis, the inaccuracies of positioning increase. In turn, the accuracy of positioning the cargo is higher for the first case when the operator of the crane is located at a distance of $x_{Op}$ = −3 ÷ 0.2 m from the vehicle. A determining influence on this is exerted by the higher accuracy of the unidirectional angular positioning of the crane column. The crane operator is able to assess the angle between the crane boom and target direction (low value of $\varepsilon_{di}$ angle) more precisely.

As the operator moves away along the *x* axis, the accuracy of the cargo positioning increases for the second case. It is easier for the operator to estimate the length of the retraction of the telescopic boom (higher accuracy of the unidirectional linear positioning). At the same time, however, it is more difficult for him to visually assess the angle between the crane boom and the target direction of the cargo (the inaccuracy of unidirectional angular positioning is increasing).

**Figure 13:**
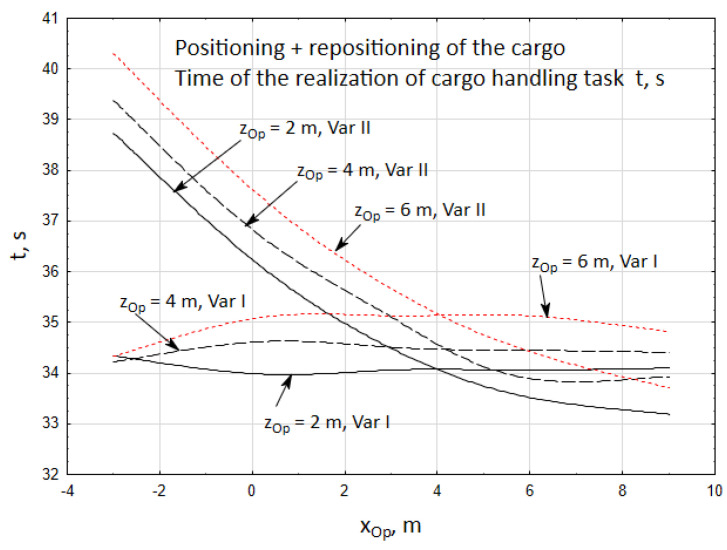
Time of the initial positioning of the cargo *t*, for two cases of the cargo handling task being realized.

Simultaneously, it was observed that the time required to complete the cargo handling task for the first case only slightly depends on the operator’s position and falls within a narrow time frame of *t* = 34 ÷ 35.2 s (Figure 13).

In the second case, however, this time is much longer since the operator of the crane is positioned at a distance of $x_{Op}=-$3 ÷ 2 m. The increased unloading time results from a high inaccuracy of unidirectional positioning of the telescopic boom moving to a significant distance with the velocity of $\dot{\delta }t$ = 0.3 m/s.

**Figure 14:**
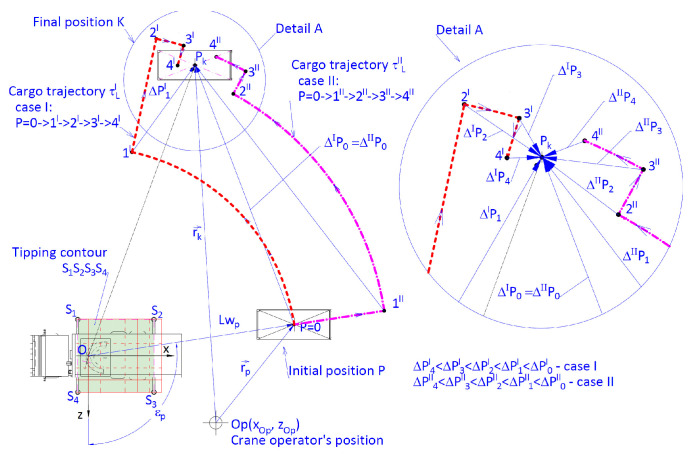
Diagram of the working cycle of the crane for assessing the accuracy of positioning the cargo in intermediate positions (1${}^{I}$, 2${}^{I}$, 3${}^{I}$, 4${}^{I}$, 5${}^{I}$, 6${}^{I}$—case I and 1${}^{\mathrm{II}}$, 2${}^{\mathrm{II}}$, 3${}^{\mathrm{II}}$, 4${}^{\mathrm{II}}$, 5${}^{\mathrm{II}}$, 6${}^{\mathrm{II}}$—case II) in relation to the final position depending on the crane operator’s position—Op for two cases of the cargo handling task.

In the next example, the cargo handling task presented in Figure 14 was being realized in two stages. The first stage consisted in three sequential movements conducted with possibly high velocities (initial positioning). In the second stage, the working movements of the crane were purposefully slowed down three times (repositioning), until the estimated accuracy of placing the cargo in the target position was obtained. For comparative purposes, we analysed two cases, in which the working movement consisted of six sequential movements (Table 3), with parameters listed in Table 4.

**Figure 15:**
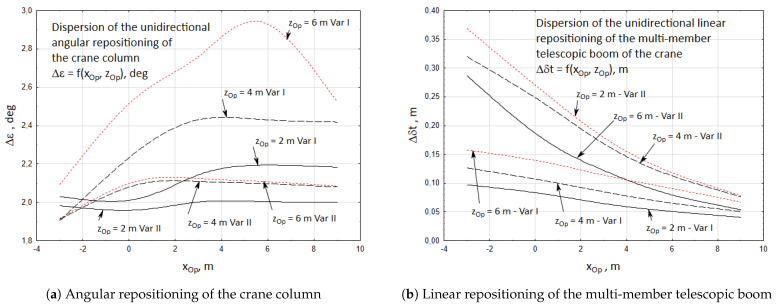
Accuracy of the unidirectional positioning of the cargo.

The graphs presented in Figure 15 show the comparison of the accuracy of the unidirectional angular (Figure 15a) and linear (Figure 15b) repositioning. The magnitudes of these inaccuracies were inserted as the input movement parameters of the simulation model.

**Figure 16:**
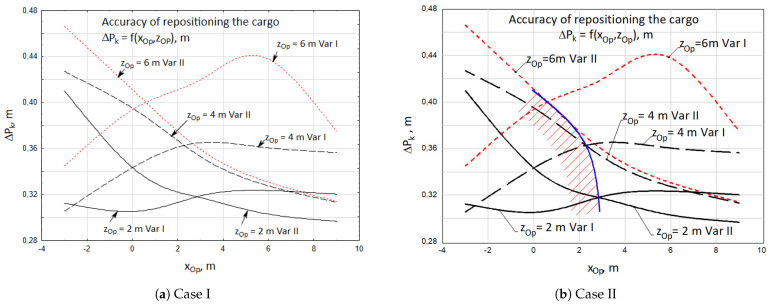
Accuracy of repositioning the cargo $\Delta P_{k}$, for two cases of the realized cargo handling task.

The obtained results indicate (Figure 16) that the accuracy of the final positioning of the cargo is significantly higher than for the initial positioning. For both cases the summary maximum dispersion of the cargo falls within the range of $\Delta P_{k}$ = 0.29 ÷ 0.465 m. However, higher accuracies of positioning towards the *x* axis and towards the *z* axis were obtained when the crane operator is located in the zone $x_{Op}$ = −2.5 ÷ 3.5 m.

The desired position of the cargo, obtained at the repositioning stage, results from the higher precision of estimating the cargo position by the operator and slowing down the movements of the working members of the crane.

**Figure 17:**
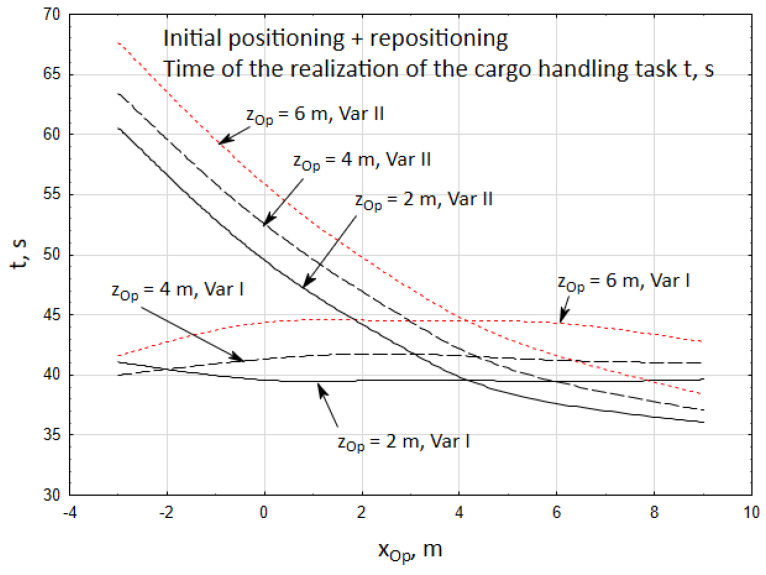
Time of the realization of the cargo handling task *t*, *s*.

At the same time it was observed that the time of the realization of the cargo handling task in the first case only slightly depends on the position of the operator and it falls within a narrow time frame of *t* = 39 ÷ 44 s (Figure 17). However, in the second case it is much longer when the crane operator is $x_{Op}$ = −3 ÷ 2.5 m away from the crane. Extending the time of unloading the cargo results from the high inaccuracy of unidirectional positioning of the telescopic boom moving to a significant distance with the velocity of $\dot{\delta }t$ = 0.1 m/s.

**Figure 18:**
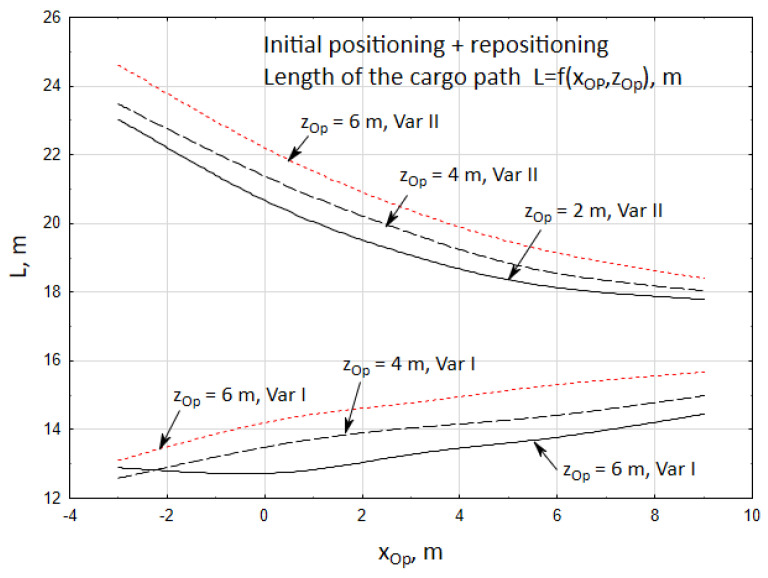
Length of the path of the carried cargo *L*, *s*.

In turn, Figure 18 presents the changes in the length of the cargo way for two cases of the cargo handling task. The most optimum solution, from the point of view of minimizing the length of the cargo way covered by the carried cargo is case I, for whom the length of the cargo way fell within the range of *L* = 12.8 ÷ 15.7 m.

In the second case, the length of the cargo way fell within the range of *L* = 17.8 ÷ 24.6 m, so it is much longer than in case I. Hence, case I is the optimum solution. In this case, the accuracy of repositioning the cargo is approximate in both variants, while the time of the cargo handling task realization is generally shorter when the crane operator is closer to the vehicle. At the same time, independently of the operator’s position, the length of the way *L* of the carried cargo is significantly shorter in the first case.

## 5. Assessment of the Accuracy of Positioning and Application Conclusions

**Figure 19:**
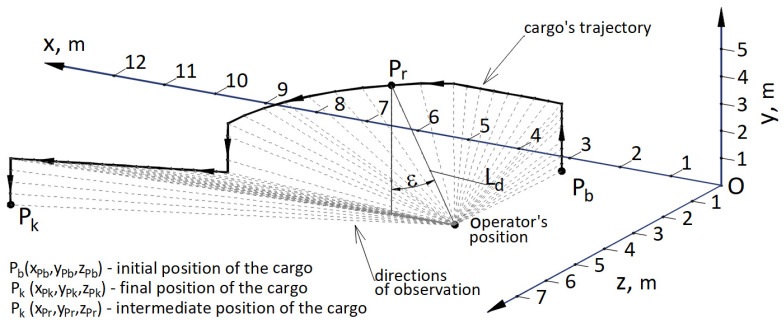
Calculation diagram for the indicator of the accuracy of positioning $W_{sk}$, where: Pb($x_{Pb}$, $y_{Pb}$, $z_{Pb}$)—initial position of the cargo, Pr($x_{Pr}$, $y_{Pr}$, $z_{Pr}$)—intermediate position of the cargo, Pk($x_{Pk}$, $y_{Pk}$, $z_{Pk}$)—final position of the cargo.

**Figure 20:**
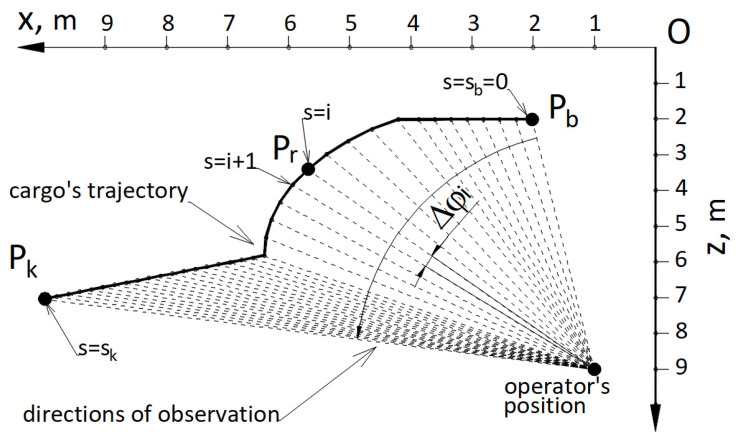
Calculation diagram for the indicator of the accuracy of positioning $W_{3}$.

The authors developed an indicator of the accuracy of positioning $W_{sk}$ depending on the position Op($x_{Op}$, $y_{Op}$, $z_{Op}$) of the operator or the video camera and the cargo movement trajectory; in the domain of the discrete values the indicator depends on the coordinates of points *P*(*x*, *y*, *z*) describing the subsequent location of the centre (base) of the cargo. In the initial position, the coordinates of the centre of the cargo are: Pb($x_{Pb}$, $y_{Pb}$, $z_{Pb}$), in the final position they are: Pk($x_{Pk}$, $y_{Pk}$, $z_{Pk}$), while in the intermediate positions they are: Pr($x_{Pr}$, $y_{Pr}$, $z_{Pr}$). The indicator depends on three factors:

- Indicator $W_{1}$ of the distance of the cargo $L_{d}$ from the observer (camera), Figure 19, can be calculated from the Formula (24):
  (24) $$W_{1}=1-L_{d}\left(x_{pr},y_{pr},z_{pr}\right)/L_{d_{max}}$$
  where: $L_{d}$ = $\sqrt{{\left(x_{Pr}-x_{Op}\right)}^{2}+{\left(y_{Pr}-y_{Op}\right)}^{2}+{\left(z_{Pr}-z_{Op}\right)}^{2}}$—distance between the cargo and the camera (observer) position, $L_{d_{max}}$—maximum distance possible to achieve between the cargo and the beginning of the system of coordinates Oxyz, which equals the value of the outreach of the cargo handling crane.
- Indicator $W_{2}$, calculated with Formula (25), Figure 19, determining the influence of the observation direction angle ε produced with a horizontal plane located at the height of the camera operator’s eyes.
  (25) $$W_{2}=\cos\varepsilon$$
- Indicator $W_{3}$, calculated with Formula (26)—Figure 20, determining the velocity of the change of the angle dφ in view of the observation directions projected onto the horizontal plane Oxz.
  (26) $$W_{3}=\left|\sin\left(d\varphi \right)\right|$$
  where: dφ = $\Delta \varphi /\Delta_{t_{s}}$; Δφ—an increase of the observation angle in projection to a horizontal plane Oxz in the subsequent step *s* of cargo dislocation; $\Delta_{t_{s}}$—duration of the subsequent step of cargo dislocation; $s=s_{b}=0$, …, s=i, s=i+1, …, $s=s_{k}$; $s_{b}$—initial step, $s_{k}$—final step.

The value of the indicator of the accuracy of positioning, depending on the indicators $W_{1}$, $W_{2}$, and $W_{3}$ is expressed with the Formula (27):

(27) $$W_{sk}=W_{1}\cdot \left(W_{2}+W_{3}\right)/2=(1-L_{d}/L_{d_{max}}\cdot (\cos\varepsilon +|\sin\left(d\varphi \right)\left|)\right)$$

**Figure 21:**
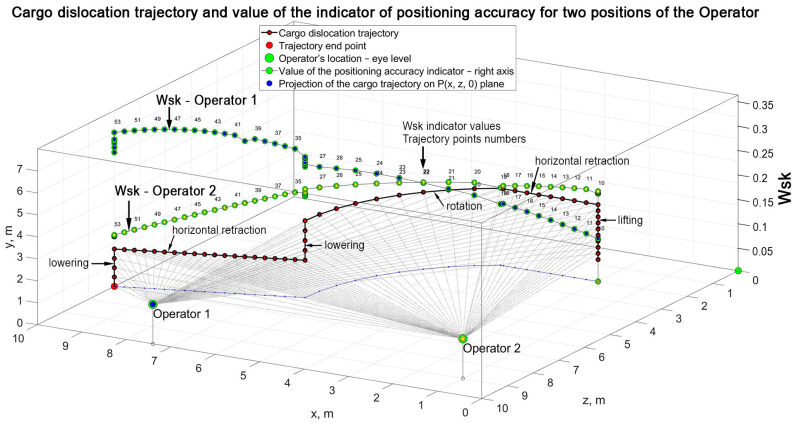
Cargo dislocation trajectory and value of the indicator of the positioning accuracy for two positions of the operator. Shaping of the values of the indicator of the accuracy of positioning $W_{sk}$.

Example results of the numerical calculations processed in MatLab software have been shown in Figure 21. The following sequence of the movement of the crane working members was adopted for the calculations (Table 5):

The values of the positioning accuracy indicator $W_{sk}$ change along the trajectory for a constant location of the operator Op($x_{Op}$, $y_{Op}$, $z_{Op}$)—a video camera. The positioning accuracy indicators $W_{sk}$ assume the values within the <0,1> range, which are reversely proportional to the increase of the distance $L_{d}$ between the operator and temporary location of cargo Pr($x_{Pr}$, $y_{Pr}$, $z_{Pr}$) and to the increase of the observation angle ε. A decrease of the $W_{sk}$ indicator is also reversely proportional for the decreasing velocities of changes dφ of the observation angle Δφ at the constant cargo movement velocity and, particularly, when the cargo approaches the operator and also in a situation when the observation angle Δφ remains unchanged.

The highest favourable value of the indicator of the accuracy of positioning $W_{sk}$ approximated to the value 1 is possible to obtain for all the points within trajectory Pr in the case of a movable location of the crane operator (or video camera) both in terms of height and the location in the horizontal plane $O_{r}x_{r}z_{r}$, on which the centre (basis) of the cargo is located. The distance $L_{d}$ can be decreased by placing a video camera near the base of the cargo. As a result, a decrease of the value of ε angle is obtained, too. The highest value of the velocity of the change of the observation angle dφ can be obtained when the axis of the camera is vertical and the ground contains recognizable elements of topography. Multiple-use markers placed on the ground in the crane operation zone can be used for this purpose.

## 6. Summary

The authors of this article conducted a quantitative assessment of the cargo positioning accuracy in a realized cargo handling task, according to a proposed methodology using an integrated CAD/CAE system. Numerical calculations were conducted in compliance with a developed mathematical model along with simulation research, which allowed the authors to analyse the influence of the order and range of the sequential dislocations of the crane’s working members on the accuracy of the final positioning, with simultaneous minimization of the movement duration and minimization of the way covered by the cargo.

An initial analysis conducted by the authors allowed them to determine the influence of the accuracy of unidirectional angular positioning of the crane column and unidirectional linear positioning of the telescopic boom on the dispersion of the cargo positioning inaccuracy. It was considered along the particular axes of the absolute system of coordinates Oxyz associated with the crane base, as well as summary position of the cargo in relations to the target position. The research included the realization of the initial positioning in which the particular movements of the working members were conducted with high working linear and angular velocities. This is the reason why the obtained results of the positioning errors are quite significant.

The second case shows how the location of the crane operator conducting a visual assessment of the cargo position influences the inaccuracy of unidirectional positioning of the crane’s working members and, consequently, the accuracy of the initial cargo positioning. The research was conducted for two cases in which the order of sequential movements was changed. It was observed that the accuracy of cargo positioning, for both cases, is higher when the operator is closer to the vehicle towards the z axis. In turn, it was observed that the accuracy of cargo positioning along the axis of the vehicle (*x* axis), is higher for the first case when the operator is closer to the vehicle’s cab.

As the operator moves away along the *x* axis, the accuracy of cargo positioning increases for the second case. It is easier for the operator to estimate the length of the telescopic boom’s retraction (increased accuracy of unidirectional linear positioning). However, it is more difficult for the operator to visually asses the angle between the crane boom and the target direction of the cargo (there is an increase in the inaccuracy of the unidirectional angular positioning).

In the other example, the cargo handling task was conducted in two stages. The first stage included three sequential movements with possibly high velocities (initial positioning). However, in the second stage, the movements of the working members were purposefully slowed down three times (repositioning) until the assumed accuracy of positioning the cargo in the target position was achieved. The obtained results show that the accuracy of the final positioning of the cargo is much higher than for the initial positioning. The desired positioning of the cargo obtained in the repositioning stage results from the higher accuracy of estimating the position of the cargo by the operator. This increased accuracy results from slowing down the working movements of the crane and from the shorter distance of the cargo from the target position.

At the same time it was observed that the time of the realization of the cargo handling task for the first case only slightly depends on the operator’s position and falls within a narrow time frame. However, in the second case it can be significantly longer, depending on the position of the operator relative to the crane system and carried cargo.

A more favourable solution, from the point of view of minimizing the distance of the carried cargo, is seen in case I. Hence, the optimum solution consists of the first case, where the accuracy of repositioning the cargo is approximate for both variants, while the time of the realization of the cargo handling task is generally shorter when the operator is closer to the vehicle. At the same time, independently of the operator’s position, the length of the carried cargo way is significantly shorter for the first case.

In this study, the authors presented the experimental results of the numerical simulation, illustrating the influence of the assumed kinematic inputs of the crane’s working members on the accuracy of positioning the cargo in the set position. It was accompanied with the graphic illustration, presenting the three-dimensional space of the dispersion of the location of the point determining the transported cargo final position.

Increasing the positioning accuracy is possible when a video camera with a variable location associated with the dislocation of the cargo is used with a vertical axis of the observation of the ground containing recognizable topographic elements, such as spatial markers, and with the system recognizing the location height by means of an integrated laser rangefinder.

## Figures and Tables

**Table 1:** Parameters of the working movements of the crane.

No.	Time (s)	Initial Position	Dislocation	
			Expected Value	Dispersion
1	0 ÷ 7.65	$\beta_{p}$ = 69.9 deg	Δβ = 19.1 deg	$\pm 3\sigma_{\beta }$ = 0
2	7.65 ÷ 12.38	$\varepsilon_{p}$ = 90 deg	Δε = −85 deg	$\pm 3\sigma_{\varepsilon }=\pm$10 deg
3	12.38 ÷ 32.38	$\delta t_{p}$ = 0.4 m	Δδt = 6 m	$\pm 3\sigma_{\delta t}=\pm$1 m
4	32.38 ÷ 33.46	$\alpha_{p}$ = 170 deg	Δα = −5.4 deg	$\pm 3\sigma_{\alpha }$ = 0

**Table 2:** Sequences of the crane’s working movements, for two cases of the realized cargo handling task during initial positioning.

Movement Sequence *i*	Cases			
	I		II	
1	Lifting the cargo	$\Delta \beta_{1}$ = 19.1 deg	Lifting the cargo	$\Delta \beta_{1}$ = 19.1 deg
2	Rotation of the column	$\Delta \varepsilon_{2}$ = 50.0 deg	Retraction of the telescopic boom	$\Delta \delta t_{2}$ = 6 m
3	Retraction of the telescopic boom	$\Delta \delta t_{3}$ = 6 m	Rotation of the column	$\Delta \varepsilon_{3}$ = 50.0 deg
4	Lowering the cargo	$\Delta \alpha_{4}=-$10.5 deg	Lowering the cargo	$\Delta \alpha_{4}=-$10.5 deg

**Table 3:** Sequences of the working movements of the crane, for two cases of the realized cargo handling task.

Movement Sequence *i*	Cases			
	I		II	
	Coarse positioning			
1	Lifting the cargo	$\Delta \beta_{1}$	Lifting the cargo	$\Delta \beta_{1}$
2	Rotation of the column	$\Delta \varepsilon_{2}$	Retraction of the telescopic boom	$\Delta \delta t_{2}$
3	Retraction of the telescopic boom	$\Delta \delta t_{3}$	Rotation of the column	$\Delta \varepsilon_{3}$
	Repositioning			
4	Rotation of the column	$\Delta \varepsilon_{4}$	Retraction of the telescopic boom	$\Delta \delta t_{4}$
5	Retraction of the telescopic boom	$\Delta \delta t_{5}$	Rotation of the column	$\Delta \varepsilon_{5}$
6	Lowering the cargo	$\Delta \alpha_{6}$	Lowering the cargo	$\Delta \alpha_{6}$

**Table 4:** Parameters of the working movements of the crane for two cases of the cargo handling task being realized.

Movement Sequence *i*	Cases			
	I		II	
	Initial positioning			
	Dislocation	Velocity	Dislocation	Velocity
1	$\Delta \beta_{1}$ = 19.1 deg	${\dot{\beta }}_{1}$ = 2.5 deg/s	$\Delta \beta_{1}$ = 19.1 deg	${\dot{\beta }}_{1}$ = 2.5 deg/s
2	$\Delta \varepsilon_{2}$ = −50 ± 23.78 deg	${\dot{\varepsilon }}_{2}$ = 18 deg/s	$\Delta \delta t_{2}$ = 6 ± 2.218 m	$\dot{\delta }t_{2}$ = 0.3 m/s
3	$\Delta \delta t_{3}$ = 6 ± 0.539 m	$\dot{\delta }t_{3}$ = 0.3 m/s	$\Delta \varepsilon_{3}$ = −50 ± 7.29 deg	${\dot{\varepsilon }}_{3}$ = 18 deg/s
	Repositioning			
4	$\Delta \varepsilon_{4}$ = 23.78 ± 2.93 deg	${\dot{\varepsilon }}_{4}$ = 6 deg/s	$\Delta \delta t_{4}$ = 2.218 ± 0.369 m	$\dot{\delta }t_{4}$ = 0.1 m/s
5	$\Delta \delta t_{5}$ = 0.539 ± 0.158 m	$\dot{\delta }t_{5}$ = 0.1 m/s	$\Delta \varepsilon_{5}$ = 7.29 ± 2.12 deg	${\dot{\varepsilon }}_{5}$ = 6 deg/s
6	$\Delta \alpha_{6}$ = −5.4 deg	${\dot{\alpha }}_{6}$ = 5 deg/s	$\Delta \alpha_{6}$ = −5.4 deg/s	${\dot{\alpha }}_{6}$ = 5 deg/s

**Table 5:** Sequence of the movement of the crane working members.

Movement Sequence	Movement of the Working Members of the Crane	Coordinates of the Cargo Pr($x_{\mathrm{Pr}}$, $y_{\mathrm{Pr}}$, $z_{\mathrm{Pr}}$), *m*	
		Initial	Final
1	Lifting the cargo—displacement 2.5 m	2.0, 1.0, 2.0	2.0, 3.5, 2.0
2	Extending the telescopic arms to the distance of 2.4 m	2.0, 3.5, 2.0	4.4, 3.5, 2.0
3	Rotation of the crane column, by the angle of −60 deg	4.4, 3.5, 2.0	6.4, 3.5, 5.8
4	Lowering the cargo—displacement 1.5 m	6.4, 3.5, 5.8	6.4, 2.0, 5.8
5	Extending the telescopic arms to the distance of 3.8 m	6.4, 2.0, 5.8	10.0, 2.0, 7.0
6	Lowering the cargo—displacement 2.0 m	10.0, 2.0, 7.0	10.0, 0.0, 7.0

## Data Availability

Data sharing is not applicable to this article.
